# Supplementary material for: Role of Telomere Length in Survival of Patients with Idiopathic Pulmonary Fibrosis and Other Interstitial Lung Diseases
Source: Biomedicines. 2023 Dec 8;11(12):3257. doi: 10.3390/biomedicines11123257 (PMC10741059; doi:10.3390/biomedicines11123257)
Supplement: Supplementary file 1 [file biomedicines-11-03257-s001.zip › biomedicines-2758207-supplementary.pdf]

Variable selection (Cox regression model)

Independent variables analyzed

Age, sex, IPF (idiopathic pulmonary fibrosis), rheumatic disease (RD), cancer, tobacco exposure, symptoms at diagnosis, T/S ratio
Interactions: age x T/S ratio, IPF x T/S ratio, RD x T/S ratio, cancer x T/S ratio

Initial selection of independent variables

Univariate hazard regression ($p < 0.3$)

*Age, sex ,*IPF, *RD, *cancer, tobacco exposure, symptoms at diagnosis, T/S ratio,
* age x T/S ratio, *IPF x T/S ratio, *RD x T/S ratio, cancer x T/S ratio

Refinement of initial selection

1. Backward Stepwise selection. p-value for exclusion ($p \geq 0.1$)

Selected variables: IPF, RD, age x T/S ratio, RD x T/S ratio,

The initial model contemplates univariate selected variables
$p = 0.9457 \geq 0.1000$, removing IPF x T/S ratio
$p = 0.9286 \geq 0.1000$, removing Cancer
$p = 0.8516 \geq 0.1000$, removing Age
$p = 0.1562 \geq 0.1000$, removing T/S ratio

2. Forward Stepwise selection. p-value for inclusion ($p < 0.05$)

Selected variables: IPF, RD

The initial model is empty
$p = 0.0352 < 0.0500$, adding IPF
$p = 0.0367 < 0.0500$, adding RD

3. Selection of the best equation by AIC/BIC and Harrell´s C criteria

Selected variables according to best AIC/BIC: age, IPF, RD, T/S ratio, RD x T/S ratio

Harrell´s C: 0.814; AIC: 81.4; BIC: 91.7 (better AIC)

Selected variables according to best Harrell´s C: age, IPF, cancer, RD, T/S ratio, age x T/S ratio

Harrell´s C: 0.825; AIC: 88.3; BIC: 100.6 (better Harrell´s C)

Variables selected in common: age, IPF, RD, RD x T/S ratio

Diagnosis of hazard Cox model assumptions.

- Colinearity. Variance inflation factor (VIF) must be < 10. Mean VIF: 4.87

- Presence of hazard proportionality (absence of relation between Schoenfeld residuals and survival time and absence of time varying covariates (tvc)). Non-significancy means that null hypothesis H_0 for proportional hazards cannot be rejected.

- Test of proportional hazards assumption based on Schoenfield residuals.

Global test. $p = 0.912$
rho Age. $p = 0.678$
rho IPF. $p = 0.830$
rho RD. $p = 0.325$
rho T/S ratio. $p = 0.530$
rho T/S ratio x RD. $p = 0.277$

- Test of interaction terms between predictors and survival time.

Global test (*chunk test*). $p = 0.632$
tvc Age. $p = 0.711$
tvc IPF. $p = 1.00$
tvc RD. $p = 0.136$
tvc T/S ratio. $p = 0.466$
tvc T/S ratio x RD. $p = 0.118$

- Test of interaction terms between predictors and logarithmic transformation of time.

Global test (*chunk test*). $p = 0.536$
tvc Age. $p = 0.545$
tvc IPF. $p = 0.534$
tvc RD. $p = 0.121$
tvc T/S ratio. $p = 0.556$
tvc T/S ratio x RD. $p = 0.086$

- Presence of log-linear relationship between instant rate of incidence and predictors. Non-significancy supports the hypothesis of linearity.

Predictor linear square analysis $p = 0.241$

- Influent observations (likelihood displacement).

2 subject detected as influents. No errors in transcription or measures.

After deleting these patients from dataset, there were no changes in significacy of predictors, except for age which turned out to be significant (HR: 1.20; 95% CI: 1.04 to 1.38; $p = 0.013$).
